# The Effect of Sealer Application Methods on Voids Volume after Aging of Three Calcium Silicate-Based Sealers: A Micro-Computed Tomography Study

**DOI:** 10.3390/tomography8020064

**Published:** 2022-03-14

**Authors:** Amre R. Atmeh, Rakan Alharbi, Ibrahim Aljamaan, Abdulrahman Alahmari, Ashwin C. Shetty, Ahmed Jamleh, Imran Farooq

**Affiliations:** 1 Hamdan Bin Mohammed College of Dental Medicine (HBMCDM), Mohammed Bin Rashid University of Medicine and Health Sciences (MBRU), Building 14, Dubai Healthcare City, Dubai P.O. Box 505055, United Arab Emirates; 2 Department of Restorative Dental Sciences, College of Dentistry, Imam Abdulrahman Bin Faisal University, P.O. Box 1982, Dammam 34212, Saudi Arabia; ralharbi84@moh.gov.sa (R.A.); ialjamaan@moh.gov.sa (I.A.); aalahmari36@moh.gov.sa (A.A.); 3 Department of Dental Education, College of Dentistry, Imam Abdulrahman Bin Faisal University, P.O. Box 1982, Dammam 34212, Saudi Arabia; asyermal@iau.edu.sa; 4 Restorative and Prosthetic Dental Sciences, College of Dentistry, King Saud Bin Abdulaziz University for Health Sciences, Ministry of National Guard Health Affairs, P.O. Box 22490, Riyadh 11426, Saudi Arabia; jamleha@ksau-hs.edu.sa; 5 Faculty of Dentistry, University of Toronto, Toronto, ON M5G 1G6, Canada; imran.farooq@mail.utoronto.ca

**Keywords:** calcium silicate-based sealers, micro-computed tomography, root canal filling, sealer application method, single gutta-percha cone, voids volume

## Abstract

During obturation, air voids are undesirable as they may provide shelter for microorganisms or passage for fluids. This study aimed to compare the occurrence of voids between three calcium silicate-based sealers (CSBSs) (MTA-Fillapex, BioRoot-RCS, Bio-C) and the change in their volume after aging. In addition, we aimed to compare voids when using two sealer application methods: lentulo-spiral (LS) and gutta-percha (GP) cone. Thirty extracted mandibular premolars (*n* = 30) were endodontically prepared and obturated using single GP cone (SGPC) technique. Each sealer was applied to 10 teeth (*n* = 10) using LS or GP. Micro-computed tomography (micro-CT) was used to quantify the volume of root filling and voids before and after 8-week storage in a phosphate-rich medium. The percentage of root filling and voids were compared between the groups using a Mann–Whitney U test and Kruskal–Wallis test with a Bonferroni correction. Before aging, the percentages of root filling volume after obturation were comparable with no significant differences between sealers (*p* = 0.325) or application methods (*p* = 0.950). After aging, the voids’ volume increased significantly in all sealers (*p* ≤ 0.05). However, no significant differences were found between sealers (*p* = 0.302). In conclusion, voids in CSBSs may not reduce in size with aging; hence, SGPC should be carefully selected for suitable cases.

## 1. Introduction

Root canal treatment aims to prevent the occurrence, or further progress, of peri-radicular disease. This is achieved by disinfecting and sealing the root canal spaces striving to entomb microorganisms that survived chemo-mechanical preparation [1]. This exhibits the pivotal role of obturation in determining the treatment outcome by providing a proper seal for the prepared root canal system [2,3]. Such a seal must isolate the remaining pathogens isolated from nutritious fluids that may percolate through permeable spaces and voids in the filled canal [4]. Root filling voids may present as open (connected to the outer environment) or closed (isolated within the root filling) [5,6]. So far, no conclusive evidence has been provided to support the superiority of any of the available root canal filling materials or techniques [7].

In recent years, calcium silicate-based sealers (CSBSs) have gained growing popularity [8]. This is partly due to the biocompatible and bioactive properties of this family of materials that allow them to exchange ions and interact with the surrounding environment including dentin [9]. For an endodontic sealer, this could be beneficial as the deposited minerals due to ionic exchange can occupy more space to compensate for the voids created during the process [10,11,12,13]. Hence, some studies used physiological fluids for aging CSBSs to simulate the ion exchange and potential mineral deposition that may take place in situ [14,15,16]. CSBS are available in a range of products varying in composition, properties, and presentation [17,18]. BioRoot RSC sealer (Septodont, Saint Maur des Fosses, France) is a CSBS presented as a powder along with its pre-proportioned liquid that is manually mixed, unlike MTA Fillapex (Angelus, Londrina, Brazil), which is presented in two manually mixed pastes. Bio-C Sealer (Angelus, Londrina, Brazil), on the contrary, is a pre-mixed sealer that is applied without the need for any manipulation. In previous reports, the preparation and mixing methods of endodontic sealers were found to affect the porosity and voids in their mixtures [19,20,21,22]. Different preparations of CSBSs were also found to influence their penetration into the dentinal tubules [23]. However, to the best of our knowledge, no correlation has been made formerly between the efficiency of different CSBSs and their preparation method before application.

With the introduction of CSBSs, the concept of single gutta-percha cone (SGPC) obturation resurfaced. With their dimensional stability, higher film thickness, and interactivity with dentin, CSBSs seemed compatible with this technique [24,25,26]. In fact, CSBSs were advocated to be used with SGPC by manufacturers. This was further supported by recent evidence reporting structural changes of CSBSs, as well as epoxy and zinc-oxide eugenol-based sealers, when exposed to heat [27,28]. Compared to cold lateral or warm vertical compaction techniques, SGPC is a simpler, more convenient, and cost-effective alternative, making it popular among dental practitioners [29,30,31]. It requires a gutta-percha (GP) cone of a size and taper which matches the final instrument used for canal preparation to be applied solely with the sealer [32]. However, evidence is still deficient about its efficiency and the quality of the seal it provides [33]. Void formation has been one of the most reported drawbacks of SGPC considering the higher thickness of the sealer that needs to be applied [33]. Another factor related to voids incidence is the sealer application method. Using epoxy resin-based sealers, Guinesi et al. reported a significantly higher incidence of voids when sealers were applied using GP cones compared with Lentulo spiral (LS) or K-files [34]. However, no correlation was found with the same sealers when these application methods were used with cold lateral compaction [34,35]. To the best of our knowledge, no evaluation has been conducted previously to compare the efficiency of different sealer application methods with CSBSs using SGPC obturation.

In the field of dentistry, the use of micro-computed tomography to study various aspects related to the root canal system is increasing [36,37]. It is a non-invasive technique that provides three-dimensional assessment and can be applied quantitatively and qualitatively [38]. Accordingly, using micro-CT, the current study’s objectives were threefold: (1) To compare the root filling volume percentage between three different CSBSs that differ in their preparation (mixing) methods—a manually mixed powder and liquid, manually mixed two pastes, and a single pre-mixed paste. (2) To compare the efficiency of two sealer application methods, LS or GP. (3) To evaluate the change in voids volume within CSBSs with aging in a phosphate-rich medium.

## 2. Materials and Methods

### 2.1. Sample Preparation

Freshly extracted human mandibular first premolars (*n* = 30) were used in this study (Ethical approval ref: EA2019018 by the Scientific Research Unit). Teeth were collected from the oral surgery clinics for patients attending for extraction of lower premolars for orthodontic purposes. Based on preliminary data, the sample size was calculated with an effect size of 10% and a 7% standard deviation using a significance level of 0.05 and a power of 80% to detect a difference. The estimated sample size for each group was required to be at least 9 (PiFace, (http://homepage.stat.uiowa.edu/~rlenth/Power/, accessed: 20 December 2021)). Teeth were visually and radiographically examined to exclude those with more than one root or canal, carious lesions, unusual anatomical features, or fractures. An impression index was prepared for each tooth’s crown using 3M™ Express™ putty vinyl polysiloxane impression material (3M ESPE, Seefeld, Germany) and cube-shaped molds (10 mm × 10 mm × 10 mm) to be used for mounting teeth during micro-CT imaging in reproducible positions.

Endodontic access cavities were prepared using a high-speed handpiece and round end tapered diamond bur (ISO 199/016). The working length was determined by measuring the length of a size 10 stainless steel K-File (Mani, Utsunomiya, Tochigi, Japan) inserted until visible through the root apex, and then deducting 0.5 mm of that length. The canals were chemo-mechanically prepared using ProTaper Universal rotary NiTi file system up to size F2 (Dentsply Sirona Endodontics, Ballaigues, Switzerland) in association with 2.5% NaOCl irrigation solution applied with a 5 mL Luer-lock syringe and MonovacTM side-vented 27 ga irrigation tip (Plasdent, Pomona, CA, USA). As a final rinse, canals were irrigated with 1 mL of 17% ethylenediaminetetraacetic acid solution (EDTA) (MD-Cleanser™, Meta Biomed Co. Ltd., Cheongju City, Chungbuk, Korea) followed by 5 mL of 2.5% NaOCl along with a manual agitation using a ProTaper F2 matching GP cone (Dentsply Sirona Endodontics, Ballaigues, Switzerland). The canals were then dried with size 25 absorbent paper points (Sure-endo, Seongnam, Korea).

Prepared teeth were randomly distributed into three groups based on the sealer used (*n* = 10): BioRoot RCS (Septodont, Saint Maur des Fosses, France), MTA Fillapex (Angelus, Londrina, Brazil), and Bio-C Sealer (Angelus, Londrina, Brazil) (Table 1).

The first two sealers were mixed as per the manufacturers’ instructions, while Bio-C Sealer was ready to use without mixing. In each group, the sealer was applied into the canals using either a ProTaper F2 GP cone (*n* = 5) or a size 25 Lentulo spiral (LS) (*n* = 5) attached to a motor-driven slow-speed handpiece (Kavo GENTLEpower LUX 20 LP, KaVo Dental, Biberach, Germany) operated at 2000 RPM with in-and-out motion keeping the instrument insertion 5 mm shorter than the working length. Each sealer application method was randomly used with half of the samples in each group. Using LS, the sealer was loaded and applied into the canal in three consecutive loads; then, the master GP cone was inserted to the full working length. For the GP cone method, the selected master cone was loaded with the sealer, applied into the canal, gently rotated while brushing against the canal walls, and repeated three times. The excess sealer was cleaned from the access cavity, and excess coronal GP was seared using a heated endodontic plugger. All obturated teeth were stored in an incubator at 37 °C and 100% humidity for 24 h before performing the baseline obturation micro-CT imaging. The samples were then separately stored in closed containers with 15 mL of a phosphate-rich solution for 8 weeks in an incubator at 37 °C. The storage solution was prepared by mixing the ingredients (Table 2) in 1000 mL of deionized water.

The acidic pH (5.5) of the mixture was adjusted to a neutral pH by mixing aliquots of 1 M of NaOH until the required pH was obtained. The solutions were replenished every week.

### 2.2. Micro-CT Imaging

**Figure 1:**
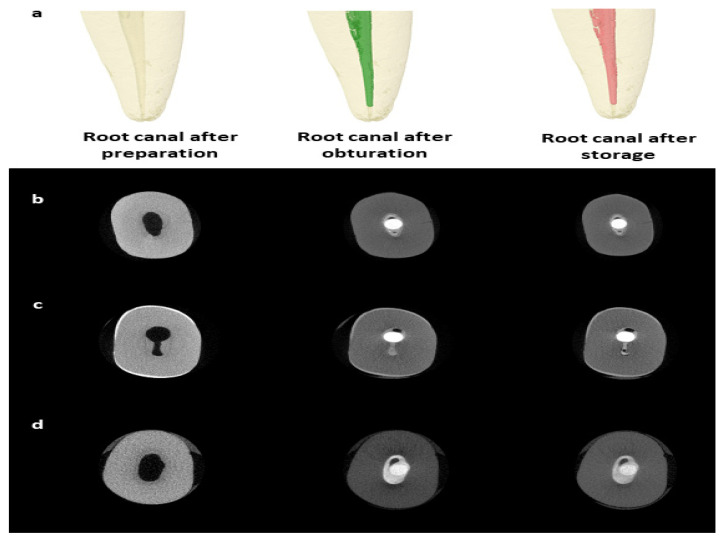
Micro-CT imaging for the teeth. Representative micro-CT reconstructed images for a tooth before obturation, post-obturation, and after 8-week storage in a phosphate-rich medium (**a**). Representative micro-CT slices for root canals filled with BioRoot RCS (**b**), MTA Fillapex (**c**), and Bio-C Sealer (**d**) with a single GP cone.

A high-resolution micro-CT system (SkyScan 1172; Bruker-microCT, Kontich, Belgium) was used with a source voltage of 89 kV, source current of 112 µA, and 5.9 s exposure time using a 360° rotational angle with a 0.6° rotational step. Raw images in Tagged Image File Format (TIFF) were produced with a pixel size of 13.73 µm and a frame averaging of two. The images were reconstructed using NRecon software (version 1.6.4.8, Skyscan, Bruker-microCT, Kontich, Belgium) from a total of 1333 slices. The reconstructed images were processed using a Gaussian kernel and ring artifact correction of 6 with 20% beam hardening correction. A standard binarization process was applied to identify and measure the voids volume, with any area falling below the threshold value of 96. This value was selected after evaluating different values and found to be the most suitable to differentiate between the root filling, dentin, and space. The micro-CT images of the teeth were obtained and reconstructed after canal preparation, post-obturation, and after 8-week storage in a phosphate-rich solution (Figure 1).

From the reconstructed images, the volumes of prepared canals (C), root filling (F), and voids (V) were measured using the following equation:

Vx = C − Fx

V1 and F1 are the void and root filling volumes (mm^3^) before storage, respectively, while V2 and F2 are the void and root filling volumes after storage. The change in void volume after storage (V3) was calculated as follows:

V3 = V2 − V1

While the percentage of change in void volume after storage (V%) was calculated as follows:

V% = (V3/V1) × 100%

### 2.3. Statistical Analysis

Data were analyzed using Statistical Package for Social Sciences (IBM SPSS Statistics for Windows, Version 23.0 released 2015, IBM Corp: Armonk, NY, USA). Descriptive analysis was performed, and the means and standard deviations are reported. The Shapiro–Wilk normality tests showed skewed distribution (*p* < 0.05). Hence, non-parametric tests of statistical significance were used. The Mann–Whitney U test and Kruskal–Wallis test with a Bonferroni correction (significance level set at *p* ≤ 0.017) for multiple comparisons (where necessary) were used for independent samples. The Wilcoxon signed-rank test was used for paired samples. A *p*-value of ≤0.05 was considered statistically significant.

## 3. Results

**Figure 2:**
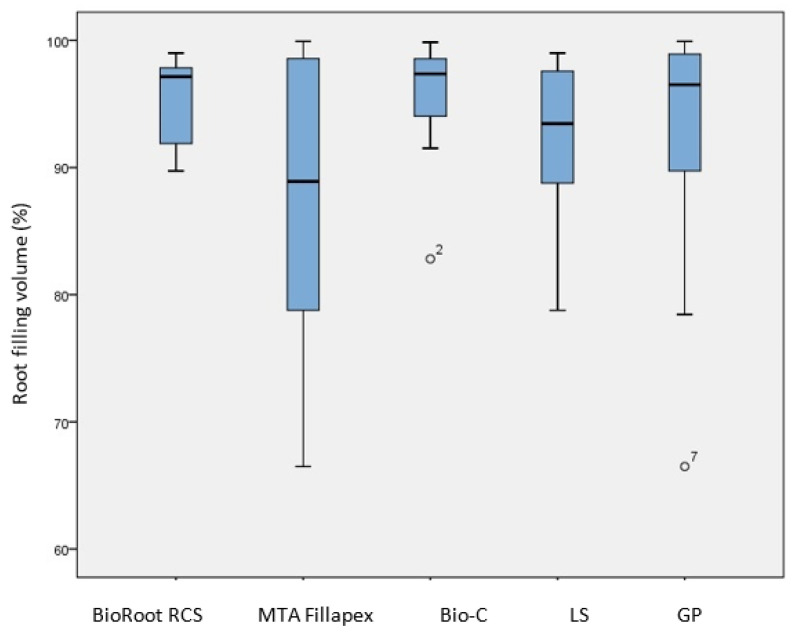
Comparison of the percentage of root filling after single GP cone obturation with different CSBSs and different sealer application methods (LS and GP). No significant differences were found between any of the groups (*p* > 0.05).

The mean ± SD of the root filling percentage is presented in Figure 2.

The average percentage for all the sealers was (92.94% ± 7.98). The highest percentage was achieved by BioRoot RCS (95.61% ± 3.46), followed by Bio-C Sealer (95.32% ± 5.14) and MTA Fillapex (87.87% ± 11.13). Although there were mean differences between the sealers, the Kruskal–Wallis test showed no statistically significant differences (*p* = 0.325). Comparing the percentage of root filling volume between the two sealer application methods (Figure 2), it was slightly higher when the LS was used. However, no statistically significant difference was found based on the Mann–Whitney U test (*p* = 0.950).

**Figure 3:**
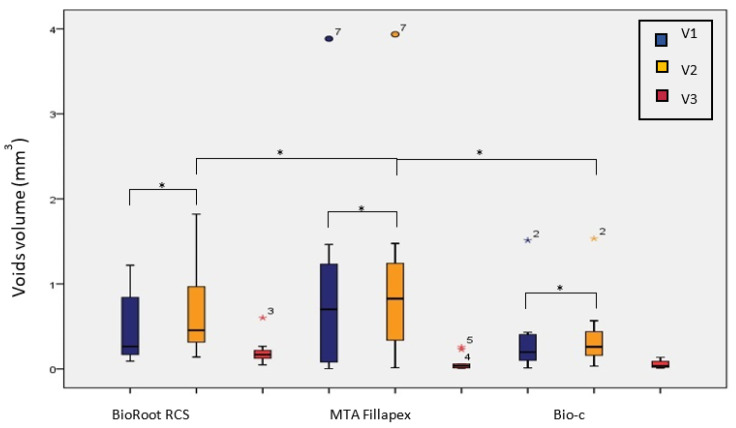
Voids volume after SGPC obturation with different CSBSs before (V1) and after (V2) storage with the volume of change (V3). (*) indicates statistically significant difference (*p* ≤ 0.05).

In all the sealers, the voids volume was significantly higher after storage (V_2_) (*p* < 0.05) based on the Wilcoxon signed-rank test (Figure 3).

The highest change in the voids volume (V3) was in BioRoot RCS (0.20 mL ± 0.15), followed by MTA Fillapex (0.07 mL ± 0.09), and the least was found in Bio-C Sealer (0.05 mL ± 0.04). These differences were statistically significant using the Kruskal–Wallis test (*p* < 0.05) (Table 3).

A Mann–Whitney U post hoc test with Bonferroni correction revealed statistically significant differences between MTA Fillapex and BioRoot RCS (*p* < 0.017) and between Bio-C and BioRoot RCS (*p* < 0.017) (Table 3). Looking at the percentage of change in the voids volume after storage (V%), the highest percentage of change was in MTA Fillapex (113.82%), followed by Bio-C (75.71%) and BioRoot RCS (63.49%) (Table 3). However, the changes were not statistically significant (*p* > 0.05) based on the Kruskal–Wallis test.

## 4. Discussion

The volumetric assessment of root filling materials is of great importance when evaluating their performance. This is principally due to the tight relation between their efficiency to fill and seal root canal spaces and the treatment’s successful outcome [39]. In this context, using micro-CT imaging has helped significantly with its unique capabilities, allowing for the indirect assessment of the root filling in relation to the canal space in a 3D configuration [40]. Adding to that, the imaged samples’ preservation makes imaging a suitable tool to observe volumetric changes in the specimens [41]. Hence, it was the instrument of choice to observe changes in the voids volume of tested root filling materials before and after storage using the same teeth with precise repositioning.

Based on our findings, no correlation was made between the sealer preparation (mixing) method and its root filling efficiency, and the occurrence of voids (Figure 2). In earlier studies that evaluated the effect of mixing methods on calcium silicate cements, no effect was found on the cements’ porosity [42] or dimensional stability [43] using micro-CT imaging, or rheological properties [44] using the glass slabs test. The mean percentage of root filling achieved for all sealers was (92.94% ± 7.98), which is comparable to previous studies [45,46,47] that used micro-CT, although higher root filling percentages were reported in other studies [48,49,50,51]. Compared with other root filling techniques, no significant differences in the percentage of voids were reported with SGPC when used with CSBSs and assessed using micro-CT [45,49,50,51]. The root filling percentage of CSBS was also comparable to other types of sealers, such as epoxy resin-based sealers, when used in this technique and assessed similarly [19,51]. Hence, it could be suggested that CSBSs would be suitable to use with SGPC and may provide a comparable root filing quality to other obturation techniques and sealers. However, they may not offer the optimal obturation efficiency ideally sought.

When CSBSs are immersed in phosphate-rich media, phosphate ions can precipitate in association with calcium ions produced by the sealer to form apatite minerals. Such deposition may take place within the voids, and hence is expected to reduce the voids volume over time [52]. Although this was reported by Gandolfi et al. studying the change in the voids volume of MTA Flow after 6-month storage in Hank’s balanced salt solution (HBSS) using micro-CT [14], the change was not significant. Our results, however, suggest the opposite, with a significant increase in the voids volume after storage in a phosphate-rich medium for all the sealers (Figure 3). Similar results were reported by a few other studies using micro-CT to assess different CSBSs [15,16], but without significant changes.

The greatest change in the voids volume was in BioRoot RCS followed by MTA Fillapex and then Bio-C sealer. However, when considering the change as a percentage to the original voids volume (Table 3), the greatest change was associated with MTA Fillapex, followed by Bio-C sealer, and the least was observed in BioRoot RCS. Such an increase in the voids volume could be attributed to the nature of these sealers, where their solubility may exceed the mineral formation rate. The relatively higher soluble nature of CSBSs has been reported before [53]. Poggio et al. reported high solubility values for BioRoot RCS when matched with other sealers [54]. In another study, it was found that both BioRoot RCS and MTA Fillapex are highly soluble and produce greater porosities [52]. The high solubility of MTA Fillapex was also reported with longer storage durations in a few studies [53,55]. Previously, Zorden-Bronzel et al. reported in an in vitro study that Bio-C sealer has higher solubility rates according to the regulations set by ISO 6876 standard [56]. Such behavior of CSBSs is expected considering their bioactive properties mediated by the dissolution of calcium silicate components and ion exchange with the surrounding environment [57]. Further studies are still needed to understand the performance of these sealers as root filling materials, comparing their solubility to their capability of apatite formation.

In terms of the efficiency of sealer application methods, no significant difference was found between the LS and GP cone. This is comparable to previous studies that reported no significant differences between the two methods using a stereomicroscope to assess the quality of the root canal fillings after root sectioning [37,38,58]. In an earlier study, Kahn et al. reported that the LS and Max-I-Probe Delivery System were the most effective in the application of endodontic sealers, with the least effective being the paper-points and K-files, to which our results are comparable [59]. Studies by Nabavizadeh et al. and Wiemann and Wilcox also reported no statistically significant differences upon comparing the efficiency of different sealer application methods (LS, GP cone, K-file, and ultrasonic files) when assessed using a stereomicroscope [58,60]. Using a confocal laser scanning microscopy, Dash et al. reported significantly higher penetration of the endodontic sealer when applied using LS compared to ultrasonic files [61]. Based on this, it can be suggested that the application method (LS or GP) may not affect the efficiency of obturation using CSBSs and SGPC.

Compared to other sealers, CSBSs exhibit minimal dimensional changes that may allow them to provide a better seal for the root canal system [62]. However, using SGPC obturation involves a higher amount of the sealer in the root filling, regardless of its type [63]. This puts more reliance on the sealer to fill the root canal space, which increases the chance of voids to occur. Furthermore, the GP itself could be also affected by the chemical nature of the sealer. Exposing GP to eugenol can lead to an expansion of the cones [64,65]. On the contrary, in a recently published study, it was reported that GP could also be affected by alkaline conditions that mimic the alkaline pH of CSBSs, causing them to shrink [66]. Hence, CSBSs could be suitable for SGPC technique; however, this obturation technique should be used for selected cases in which sealer thickness can be maintained to the minimum. A GP cone matching the size and shape of a small, prepared canal would be the most desirable, although it may not offer the optimal obturation efficiency that is ideally sought.

One of the limitations of our study was it’s in vitro nature. The real in vivo oral environment is dynamic and offers more aggressive challenges, so a similar study could yield different results under in vivo settings. Another limitation was the number of samples used and the range of sealers tested. Including more types of sealers would provide a better understanding of the differences in their performance over time. The other limitation was the measurement of the voids volume by only one analytical method, i.e., the micro-CT technique. Performing volumetric measurements using micro-CT analysis should be carefully interpreted, as factors such as voxel size, image-processing software, and the radiopacity of the material may influence the measurements [67]. Analyzing images with 13.73 µm pixel size means that a minor detectable void was around this value, which is very close to the minimal volume of air voids [68]. However, factors other than imaging resolution such as binarization and threshold setting could be crucial in determining the sensitivity of void detection and volumetric measurement; hence, chances of error cannot be eliminated.

## 5. Conclusions

Within the limitations of the current study, sealer preparation and application methods into the canal did not seem to affect the quality of the root filling when performed with SGPC. All tested CSBSs exhibited a significant increase in their voids volume after 2-month storage in a phosphate-rich medium. This refutes the suggestion that voids in CSBSs could reduce over time with mineral deposition. Therefore, SGPC obturation should be carefully selected, considering the shape and size of the canal to minimize the occurrence of voids.

## Figures and Tables

**Table 1:** Details about the endodontic sealers tested in this study.

Sealer	Presentation	Composition	LOT
BioRoot RCS	Manually mixed powder and pre-portioned liquid ampules	**Powder**: Tricalcium silicate and zirconium oxide. **Liquid**: Aqueous solution of calcium chloride and polycarboxylate.	B17882
MTA Fillapex	Manually mixed two pastes.	**Base paste**: Salicylate resin, natural resin, calcium tungstate, nanoparticulated silica, and pigments. **Catalyst paste**: Diluting resin, tri-calcium silicate, di-calcium silicate, calcium oxide, tri-calcium aluminate, nanoparticulated silica, and pigments.	101,430
Bio-C Sealer	Injectable pre-mixed paste	Calcium silicates, calcium aluminates, calcium oxide, zirconium oxide, ferric oxide, silicon dioxide, and thickening agent.	101,527

**Table 2:** Composition of the phosphate-rich storage solution.

S. No.	Ingredient	Quantity/1000 mL
1.	NaCl	0.400 g
2.	KCl	0.400 g
3.	NaH_2_PO_4_·H_2_O	0.690 g
4.	CaCl_2_·H_2_O	0.790 g
5.	Na_2_S·9H_2_O	0.005 g

**Table 3:** The mean change in voids volume (V3) and mean percentage of change (V%) for each sealer after storage.

Sealer Type	V_3_ (SD)	V_%_ (SD)
BioRoot RCS	0.20 ^a^ (0.15)	63.49 ^a^ (38.37)
MTA Fillapex	0.07 ^b^ (0.09)	113.82 ^a^ (197.90)
Bio-C Sealer	0.05 ^b^ (0.04)	75.71 ^a^ (133.39)

Values in the same column with the same superscript letter indicate no statistically significant difference (*p* > 0.05).

## Data Availability

All the data are contained within the article.
